# Role of the Microenvironment in Gastrointestinal Tumors

**DOI:** 10.1155/2019/2153413

**Published:** 2019-12-11

**Authors:** Nathaniel Weygant, Yang Ge, C. Benedikt Westphalen, Wen Wee Ma, Kenneth J. Vega

**Affiliations:** ^1^Academy of Integrative Medicine, Fujian University of Traditional Chinese Medicine, Fuzhou, China; ^2^Fujian Key Laboratory of Integrative Medicine in Geriatrics, Fujian University of Traditional Chinese Medicine, Fuzhou, China; ^3^Department of Oncology, Beijing Chao-Yang Hospital, Capital Medical University, Beijing, China; ^4^Department of Oncology, Ludwig Maximillian University of Munich, Munich, Germany; ^5^Department of Oncology, Mayo Clinic, Minneapolis, MN, USA; ^6^Department of Gastroenterology, Augusta University, Augusta, GA, USA

The gastrointestinal (GI) tract maintains a complex environment with diverse epithelial and nonepithelial cell types that regulate discrete and distant processes. Pathogens such as *H. pylori* and inflammatory conditions such as colitis, Barrett's esophagus, and pancreatitis are linked to GI cancers, and recent studies have shown that a targeted mutation in a specific cell type with or without inflammation can be sufficient to initiate cancer. Recent studies using next-generation sequencing to determine molecular subtypes have expanded our understanding of GI cancers, and efforts to go beyond basic pathological assessment including multigene biomarkers, machine learning algorithms, and organoid drug screens are underway with promising results. For therapy, nucleoside analogs, targeted inhibitors, and monoclonal antibodies have demonstrated positive but often limited results in the clinic. New therapies such as anti-PD1 immunotherapy have thus far shown benefit in subsets of patients such as those with microsatellite instability-high tumors, but have not yet delivered the transformational results seen in other cancers. In order to develop effective new therapies for GI cancers and accurately classify patient risk, an improved knowledge of molecular signaling and cell-cell interactions within the tumor microenvironment (TME) is needed. Importantly, in the current clinical context, a more complete understanding of how TME components modulate resistance to therapy and antitumor immunity will be fundamental to improving patient treatment options and outcomes. This special issue seeks to improve understanding of the molecular, cellular, and pathological characteristics of the TME in GI cancers.

In the paper by D. Qu et al., differential activities of cancer stem cell marker doublecortin-like kinase 1's isoforms in pancreatic cancer are described. Importantly, they confirm previous findings that DCLK1 can be coimmunoprecipitated with KRAS and demonstrate that DCLK1 is capable of activating RAS. They support these findings with molecular, bioinformatic, and functional analyses of downstream pathways PI3K/AKT/MTOR and demonstrate the therapeutic use of DCLK1 monoclonal antibody using *in vivo* mouse models. These results further elucidate the functional mechanisms of an important GI CSC marker. C. He et al. also focused on pancreatic cancer and evaluated the effect of irreversible electroporation on immunologic characteristics in patients with locally advanced disease. Their clinical findings demonstrate a prognostic value for CD8+ T cells in this context. They conclude that this may have value as a prognostic tool in pancreatic cancer.

Md. N. Uddin et al., H. Sun et al., and C. Just et al. presented studies concerning the development of prognostic biomarkers for GI cancers. Briefly, Md. N. Uddin et al. used a meta-analysis procedure to develop a colon tumor stroma transcriptional signature. This signature was prognostic in CRC and CD8+ T cells, and prooncogenic signaling pathways were also enriched in colon tumor stroma. H. Sun et al. investigated the prognostic potential for GLIS2 in gastric cancer. In the context of radiotherapy, low expression of GLIS2 predicted notable radiosensitivity, which might find use in improving the precision of gastric cancer radiotherapy. Whereas Md. N. Uddin et al. and H. Sun et al. utilized meta-analysis and mRNA expression to derive their respective signatures, and C. Just et al. opted to focus on small noncoding miRNAs. They performed a retrospective study of 33 patients receiving neoadjuvant chemotherapy for esophagogastric junction adenocarcinoma using a 96-well array of miRNAs with known malignant roles. They found differential expressions of Let-7f, miR-221, miR-31, miR-191, and miR-194 in this context. These findings could enable improved selection of esophagogastric adenocarcinoma patients for neoadjuvant therapy.

Finally, in a series of review articles, D. Ayers et al., C. Bazzichetto et al., A. Righetti et al., I.-H. Ham et al., E. Pretzsch et al., L. Figueroa-Protti et al., and V. Vautrot et al. cover various topics of emerging importance in the TME. These include cytokine and chemokine signaling, tumor-stromal interactions, specific, mechanisms of metastasis, epigenetic influences, exosomal miRNAs, and immune checkpoint.

